# Comparative Population Biology and Related Gene Expression in the Beta-Cypermethrin-Resistant Strains of *Bactrocera dorsalis* (Hendel)

**DOI:** 10.3390/insects15080569

**Published:** 2024-07-26

**Authors:** Doudou Li, Langjie Chen, Xinyan Cai, Yixiang Qi, Yongyue Lu

**Affiliations:** Department of Entomology, College of Plant Protection, South China Agricultural University, Guangzhou 510642, China; lddoui@163.com (D.L.); cljrogers@163.com (L.C.); xinyan_cai@126.com (X.C.); qiyixiang19880922@163.com (Y.Q.)

**Keywords:** *Bactrocera dorsalis*, beta-cypermethrin, target genes, resistant strain

## Abstract

**Simple Summary:**

*Bactrocera dorsalis* (Hendel) (Diptera: Tephritidae) is native to tropical and subtropical regions and is a major pest of many important fruit trees and vegetables. In this study, the egg hatching rate, larval/pupal rate, pupal feathering rate, and pre-laying stage of the beta-cypermethrin-medium-resistant strain were found to be markedly lower compared to the sensitive strain. In contrast, the average adult longevity and female longevity of the high resistant strain were significantly longer than those of the sensitive strain, though the fecundity of the resistant strains was lower than that of the sensitive strain. Population growth was suppressed in the medium-resistant strain, while it was enhanced in the high resistant strain populations. In this experiment, the annotation of the Suppression Subtractive Hybridization (SSH) library revealed that the relevant genes mainly pertain to metabolic processes in biological pathways, followed by genes related to cellular processes. Regarding molecular functions, relevant genes mainly pertain to catalytic and binding functions. In terms of cellular-component-related genes, they are mainly associated with cell, organelle, and macromolecule complexes. So, the proportion of the relevant genes in the beta-cypermethrin-resistant strains of *Bactrocera dorsalis* is the same as the gene expression pattern in the organism, which suggests that the development of resistance in insects does not change the proportion of the genes.

**Abstract:**

Diptera and Lepidoptera species have the highest levels of insecticide resistance, and the mechanism of drug resistance has been studied in detoxification metabolism genes such as P450, GST, EST, and ABC. Since *Bactrocera dorsalis* are resistant to a variety of chemicals, the pattern and mechanism of resistance in *Bactrocera dorsalis* have been investigated from a variety of aspects such as detoxification metabolism genes, detoxification enzymes, intestinal symbiotic bacteria, and synergists in the world. In this study, 51 species and 149 detoxification metabolism genes were annotated in the Suppression Subtractive Hybridization (SSH) library, and 12 candidate genes related to beta-cypermethrin resistance were screened and quantitatively expressed in this library. Two genes were found to be upregulated in the egg stage, three genes in the larval stage, one gene in the pupal stage, and five genes in the adult stage, and four genes were found to be upregulated in the midgut and the malacca ducts in the midgut. The expression of *cyp6g1*, *cyp6a22*, *GST-Epsilon9*, and *Trypsin-4* genes was upregulated in resistant strains, with the most obvious upregulation occurring in the midgut and the Malpighian tubules. These results provide new insights into the study of pesticide resistance in quarantine insects.

## 1. Introduction

The *Bactrocera dorsalis* (Diptera: Tephritidae) is an important quarantine plant pest. Transcriptomic studies have revealed the prevalence of duplications in genes, explaining the diversity in the *B. dorsalis* complex, as well as the invasion of the species and its rapid adaptation and expansion [1,2,3,4]. The oriental fruit fly has a wide range of hosts, high adaptability, and strong reproductive capacity and causes serious harm. It can harm more than 600 kinds of fruits and vegetable crops such as citrus, guava, mango, pomelo, pumpkin, pepper, and others, which causes great losses to agricultural production. The damage caused by the fruit fly is mainly due to the female adults using their oviposition needles to puncture the fruit skin and lay eggs inside the fruit, causing the formation of oviposition holes and larvae hatching in the fruit for feeding. Female fruit flies can mate and lay eggs within 15 days, with the spawning period lasting up to three months [5,6,7]. Consequently, it has become one of the most important pests of fruits and vegetables in southern China [8,9]. In recent years, reports have indicated the presence of the oriental fruit fly in central China (Henan and Hunan Province), western China (Sichuan), and northern China (Liaoning Province), leading to risk assessments [10].

The major categories of organic chemical pesticides commonly used are pyrethroids, organophosphates, carbamates, and others. Since the synthesis of DDT in 1874, pest control has ushered in the age of chemical control [11]. Chemical pesticides are known for their fast efficacy, good effect, strong pertinence, and wide spectrum of insecticidal properties. Current chemical agents include both organic and inorganic chemical pesticides [12]. The main methods of controlling the oriental fruit fly include agricultural control (e.g., planting avoidance plants) [13], biological control (e.g., killing larvae by releasing parasitic wasps to lay eggs) [14], physical control (e.g., yellow board baiting, silver avoidance) [15], and chemical control (e.g., using chemical pesticides and biopesticides for control) [16]. At present, chemical control is the most commonly used and effective means of field control of the oriental fruit fly. However, the long-term use of the same type of chemical pesticide can develop resistance and pass it on to future generations. The beta-cypermethrin insecticide is still one of the main chemicals used in the control of orange fruit flies, and beta-cypermethrin has been found to develop a certain level of resistance in the control of orange fruit flies in resistance assays [17,18]. Consequently, the emergence of resistance has become a major problem to be solved in pest control. Currently, up to 80% of fruit flies are resistant to pesticides, which affects their fecundity, hatchability, pupal feathering, and longevity [19,20,21]. The emergence of insecticide resistance is mainly influenced by two factors, the environmental factors and the detoxification metabolism enzymes in the insect body [22]. In recent years, the most studied detoxification metabolism enzymes are cytochrome P450, acetyl chostrainterase, and glutathione S-transferase [23,24]. A study suggests that gut microbes enhance insecticide resistance in oriental fruit flies [25]. It has also been shown that odor-binding proteins help reduce insect susceptibility to insecticides and increase insect resistance [26]. One study used a proteomic approach to identify resistance response proteins to pyrethroid toxicity in the oriental fruit fly larvae [27].

Suppression Subtractive Hybridization (SSH) libraries are an effective means for the detection of expressed genes in organisms, allowing the identification of the differential expression profiles of a large number of genes at once, which help enrich and clone the target genes and analyze them functionally. Since the creation of this technology in 1996, it has been applied in many fields to provide accurate and highly expressed differential genes, supporting research work on resistance genes [28,29,30]. And by combining SSH technology with cDNA microarrays, Yang isolated and identified differentially expressed genes in ER-negative and -positive cell lines of breast cancer in 1999 [31]. Analysis of the SSH library of the rice variety Aganni reveals candidate gall midge resistance genes [32] and differential gene expression in gall midge-susceptible rice genotypes [33].

In this study, we investigated the population biology of beta-cypermethrin-resistant strains of the oriental fruit fly and analyzed the differences in biological and population parameters between resistant and sensitive strain. This research aims to provide reference and guidance for the management of oriental fruit fly resistance in the field. By annotating and comparatively analyzing the SSH library of beta-cypermethrin-resistant strains of the oriental fruit fly, we identified differentially or co-expressed genes related to insecticide resistance, which can provide a reference for the study of resistance genes in the oriental fruit fly.

## 2. Materials and Methods

### 2.1. Fly Strains

Sensitive strain of the oriental fruit fly (SS): These strain were harvested from Yangtao Park in Guangzhou City (Guangdong, China), and the fruit fly larvae were isolated and obtained. The larvae pupated, and after adult emergence (male and female 1:1), they were reared in an indoor (temperature: 26 ± 2 °C, humidity: 60–70%, and a 14:10 h light/dark photoperiod) 35 × 35 cm cage using artificial feeds for more than 52 generations without any exposure to insecticides. The larvae of the oriental fruit fly were reared on an artificial diet, and all adults were fed a 1:1 yeast/sugar artificial diet along with water [20].

High level of resistance to beta-cypermethrin (BC-H): The sensitive strain (LC_50_: 3.060 mg/L) was treated with beta-cypermethrin for 35 generations at intervals, determining a population with 127.38 times multiplicity of resistance (LC_50_: 389.769 mg/L).

Medium level of resistance to beta-cypermethrin (BC-M): The sensitive strain was treated with beta-cypermethrin for 35 generations at intervals, determining a population with 29.84 times multiplicity of resistance (LC_50_: 91.324 mg/L).

Low level of resistance to beta-cypermethrin (BC-M): The sensitive strain was treated with beta-cypermethrin for 5 generations at intervals, determining a population with 7.42 times multiplicity of resistance (LC_50_: 22.71 mg/L).

Beta-cypermethrin: (purity ≥ 98%, CAS:65731-84-2) The insecticide was purchased from Guangdong Liwei Chemical Industry Co., Ltd. (Guangzhou, China).

Total RNA extraction reagent: RNAiso Plus was purchased from Bao Bioengineering (Dalian) Co., Ltd. (Dalian, China) and a first-strand cDNA reverse transcription kit was obtained from Tiangen Biochemical Technology (Beijing) Co., Ltd. (Beijing, China).

Primers: The primers were synthesized by Shanghai Biotechnology Bioengineering Co., Ltd. (Shanghai, China).

### 2.2. Bioassay and Selected Methods

Toxicity determination was carried out by using the residual contact method [22]. The chemicals were prepared in acetone as 100 mg/L stock solutions. Beta-cypermethrin stock solutions were diluted to 5–6 different concentrations with acetone; 5 mL of each diluted solution was added to a separate 250 mL clean conical flask, which was gently rotated so that the solution evenly coated the inside of the flask until the acetone evaporated. Twenty adult flies (1:1 sex ratio) (3–5 days after emergence) were placed in a prepared conical flask, which was sealed with gauze that had cotton dipped in 10% honey water placed on top (six replicates per concentration). The mortality rate was observed after 24 h. The adults were gently turned over with a brush and considered dead if they could not turn back over within 30 s. If the mortality rate in the control group (acetone only) was <10%, the experiments were considered valid, and the adjusted mortality was corrected using Abbott’s formula; if the mortality rate in the control group exceeded 10%, the experiment was considered invalid and repeated.

Experiments on selected fly strains were carried out using the residual contact method. Take the medium level of resistance to beta-cypermethrin strain as an example. Adults of 3–5 days of age were treated with a median lethal concentration (LC_50_) using a population screening method, and the F_0_ generation was established as the susceptible strain (SS). The F_0_ generation was eliminated using the F_0_ LC_50_ for the F_1_ to F_3_ generations, the F_4_ generation was eliminated using the F_4_ LC_50_ for the F_4_ to F_6_ generations, the LC_50_ was re-determined every 3 generations, the current and next 2 generations were selected using the LC_50_ of the obtained agent, and so on. The high level of resistance to beta-cypermethrin strain were obtained by LC_80_ treatment.

### 2.3. Determination of Egg, Larval, and Pupal Developmental Duration, Hatching Rate, Pupal Rate, Emergence Rate, and Female-to-Male Ratio of Different Strains of the Oriental Fruit Fly

An appropriate amount of orange juice was poured into a homemade ovipositor and shaken well. It was then placed in the incubator cage at the peak of oviposition. After the adults had laid eggs for 1–2 h, the eggs were removed with a small brush. The eggs were placed into a plastic culture box with artificial feed and observed every 6 h, with the number of eggs hatched recorded each time. Three replications were set up, with 50 eggs inoculated in each replication. After the eggs were hatched, the larval development was observed and recorded at 9:00 and 21:00 every day until the larvae matured. The period from hatching to mature larvae was recorded as the larval developmental period, and the pupal rate was calculated. Healthy mature larvae of the oriental fruit fly that came out of the feed box on the same day were put into plastic cups filled with wet sand (relative moisture content of about 70%) and then observed every 6 h after reaching the pupal stage, recording the pupal developmental period, the emergence rate, and the sex ratio of adult males and females. Three replications were set up, with 50 heads in each replication.

### 2.4. Determination of Survival Rate and Fecundity of Adults of Different Strains of the Oriental Fruit Fly

Healthy adult oriental fruit flies, after one day of emergence, were transferred to insect-rearing cages and provided with water and artificial feed. Starting 5 days after emergence, an egg collector filled with orange juice was placed into the insect-rearing cage to catch eggs. The fecundity and the number of adult oriental fruit fly deaths were observed and recorded daily until all adults died. Three replications were set up, with 40 pairs of adults per replication.

### 2.5. Annotation of Insecticide-Resistant SSH Library of the Oriental Fruit Fly

Suppression Subtractive Hybridization (SSH) libraries were constructed by taking 4 females and 4 males from each of the sensitive, low, medium and high resistance strain of the oriental fruit fly and the adults of the same strain at 5 days post-feathering. The library was prepared and obtained by Jiang Jianjun [34]. The Blast2go software was used to annotate the beta-cypermethrin-resistant SSH library, with all parameters set to the default values of the software. This was supplemented by MEGA (MEGA 6.0) software to clip the sequences in the library, deduce their amino acid sequences, and perform Blast-x comparison with the NCBI web database (http://blast.ncbi.nlm.nih.gov/Blast.cgi, URL accessed on 25 June 2015). Based on the annotation and comparison results, differential and co-expressed genes were identified by comparing the beta-cypermethrin-resistant SSH libraries of the oriental fruit fly [34].

### 2.6. Extraction of RNA and Reverse Transcription of cDNA

Eggs (50 eggs), 3rd instar larvae (6 individuals), 5th day pupae (6 individuals), and adults (3 males + 3 females) 3–5 days after emergence, along with dissected adult tissues of sensitive and beta-cypermethrin-resistant strains of oriental fruit fly, were taken for RNA extraction, respectively. The tissues were ground with an automated grinder and tissue RNA was extracted using Trizol Reagent and dissolved in RNase-Free water. After extraction, RNA quality was assessed using a Nano-2000 micro-UV spectrophotometer (NanoDrop Technologies, Inc., Wilmington, DE, USA) and 1% agarose electrophoresis, respectively. cDNA synthesis was immediately carried out using a Fast Quant cDNA kit.

### 2.7. Primer Design for Real-Time PCR

Primers (Table 1) were designed for the candidate genes from resistance genes of oriental fruit flies screened from the SSH library using Primers 5.0 software and NCBI Primer-blast (http://www.ncbi.nlm.nih.gov/tools/primer-blast, URL accessed on 25 June 2015), an online primer design tool, according to the requirements for fluorescent quantitative PCR [35].

The real-time PCR system was prepared using the 20 μL system in Takara SYBR^®^ Premix Ex Taq II (TliRNaseH Plus) reagent. Reaction conditions were as follows: pre-denaturation at 95 °C for 10 min, denaturation at 95 °C for 30 s, annealing at 55 °C for 30 s, and extension at 72 °C for 30 s, and this process was repeated for 40 cycles; finally, the lysis curve process was measured at 55–95 °C. After determining that no primer non-specific amplification and primer dimerization exists by lysis curve analysis, the cDNA template was diluted into 5 concentration gradients at 1:10 (*v*/*v*) for standard curve analysis. Once the amplification efficiency of all primers was determined, real-time PCR was performed on each sample using *α-tub* as the internal reference gene. Three replicates were set up for each treatment.

## 3. Data Analysis

The 2^−ΔΔCT^ method was used to analyze the relative expression of each gene in different developmental durations and different adult tissues using α-tub as the internal reference gene. Excel version 2021 software was used to count the data and perform basic graphing [36]. Probabilistic regression analyses were performed using SPSS version 19.0 (SPSS, Inc., IBM, Armonk, NY, USA) to calculate the LC_50_ values of pesticides against fruit flies and the corresponding 95% confidence limits (CLs). The SAS software (version 9.4) was used for all other data analyses. Significance analyses of the experimental results were performed using Duncan’s multiple-range test (DMRT) or the *t*-test (with validation of normal distribution before data analysis). We set *p* < 0.05 as the threshold for statistical significance. The data generated in this study were subjected to analysis of variance (ANOVA) followed by Tukey’s or Friedman’s post hoc tests [20].

The classification of resistance levels is based on the multiplicity of resistance: <3 times are sensitive; 3–10 times is a low level of resistance; 10.1–40 times is a medium level of resistance; 40.1–160 times is a high level of resistance; and >160 times is a very high level of resistance.

## 4. Results

### 4.1. Comparison of the Developmental Duration and Survival of Eggs, Larvae, and Pupae between Beta-Cypermethrin-Resistant and Sensitive Strains of the Oriental Fruit Fly

There was no significant difference in the developmental duration of the eggs among the three strains: SS, BC-H, and BC-M, which were 45.83 h, 53.17 h, and 45.00 h, respectively. The results of hatching rate showed that the BC-H strain (93.67%) was significantly higher than the BC-M strain (82.67%). The hatching rate of the SS strain was 88.33%, which was not significantly different from both resistant strains (df = 2, F = 3.43, *p* = 0.067 > 0.05). The results of the oriental fruit fly larvae showed no significant difference in the developmental duration of the larvae of the three strains, which were 123.17 h for the SS strain, 124.50 h for the BC-H strain, and 122.67 h for the BC-M strain. The larvae of the SS and BC-H strains had significantly higher pupal rates of 95.18% and 96.66%, respectively, while the BC-M strain had a lower pupal rate of only 84.79% (df = 2, F = 5.79, *p* < 0.05). The pupal developmental duration of all three strains ranged from about 232 h to 242 h, with the SS and BC-M strains having a similar developmental duration of 231.78 h and 234.50 h, respectively. The BC-H strain had the longest developmental duration of 241.67 h. The BC-M strain had a significantly lower emergence rate (89.27%) than that of the SS strain (97.93%), while the result for the BC-H strain was 95.76%, which showed no significant differences compared with the SS and BC-M strains (df = 2, F = 3.28, *p* = 0.58 > 0.05) (Table 2).

### 4.2. Main Biological Parameters of Beta-Cypermethrin-Resistant and -Sensitive Strains of the Adult Oriental Fruit Fly

The male longevity of the three strains of the oriental fruit fly ranged from about 123 to 144 days, which presented no significant changes. Female longevity ranged from 119 to 150 days (df = 2, F = 8.38, *p* < 0.05), and the average longevity of males and females ranged from 127 to 150 days (df = 2, F = 3.91, *p* = 0.082 > 0.05). The SS strain had significantly shorter longevity than the BC-H strain, while the BC-M strain did not differ notably from either of them. There was no significant variation in fecundity among the three strains (Table 3).

**Figure 1:**
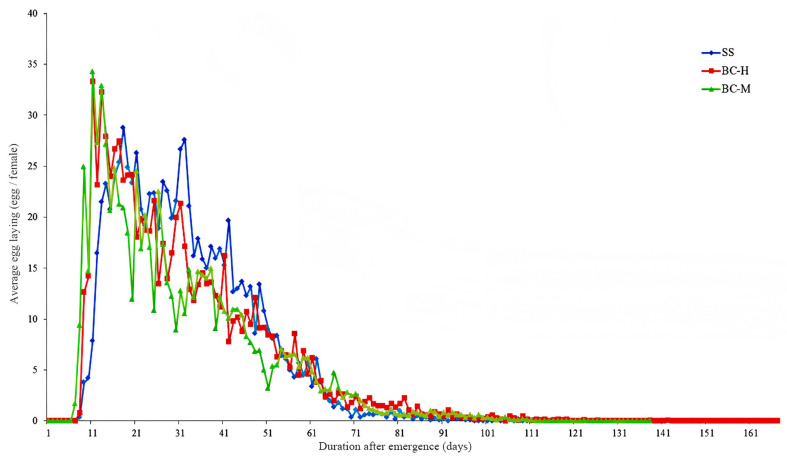
Dynamics of daily fecundity in females of beta-cypermethrin-resistant and -sensitive strains.

All strains had similar daily fecundity dynamics in females, indicating that the oviposition peak periods come in a short time after the sexual maturity of females. After the oviposition peak, fecundity decreased in a fluctuating manner (Figure 1). Compared with the SS strain (20 d), the beta-cypermethrin-resistant strains entered the oviposition peak earlier (BC-H and BC-M: 11 d), with the oviposition peak reaching 28.8 eggs/female^−1^, 34.3 eggs/female^−1^, and 33.3 eggs/female^−1^ for SS, BC-M, and BC-H, respectively. From the oviposition peak and until 50 days after emergence, the females of the SS strain showed higher fecundity, followed by those of the BC-H strain, and then the BC-M strain.

**Figure 2:**
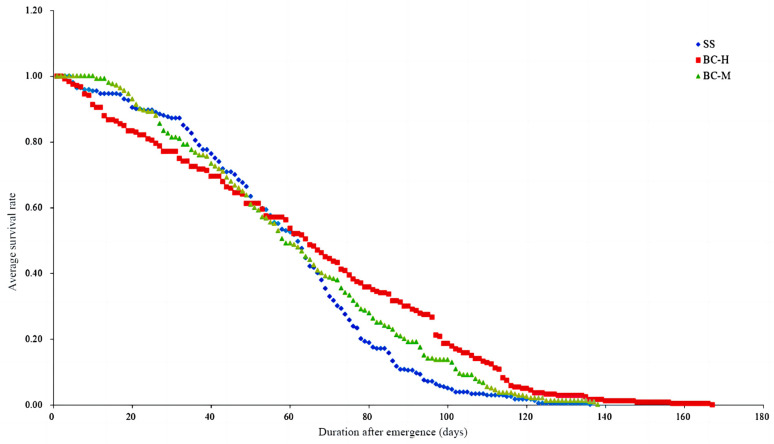
Dynamics of adult survival rates of beta-cypermethrin-resistant and -sensitive strains of the oriental fruit fly.

The survival rate of the adult oriental fruit fly of the BC-M strain was higher than that of the SS and BC-H strain from 1 day to 20 days after emergence. The SS strain had a higher rate than the resistant strains from 20 to 55 days, while the BC-H strain had a significantly lower value. After 63 days, the survival rate of the SS strain was significantly lower than that of the beta-cypermethrin-resistant strain, with that of BC-H being even higher. The survival curve of the SS strain was close to an S-shape, the curve of the BC-M strain was similar, and the survival curve of the BC-H strain was closer to linear (Figure 2).

The adult oriental fruit fly survival dynamics equations for the SS, BC-H, and BC-M strains were established (Table 4). The c-values of the equations for the three strains were 2.600, 1.734, and 2.119, respectively, which were all greater than 1 and consistent with the basic model of type I survival curves. This indicates that the majority of the adult oriental fruit flies of the three strains could reach their average longevity under the ideal laboratory environment and died mainly due to senescence.

### 4.3. Life Tables of Experimental Populations to Beta-Cypermethrin-Resistant and -Sensitive Strains of the Oriental Fruit Fly and Their Fitness

The female ratios of adults of SS, BC-M, and BC-H strains were 0.4768, 0.5014, and 0.4878, respectively, which were not significantly different. From the population trend index (I), it can be seen that the highest was the BC-H strain (I = 358.7071), followed by the SS strain (I = 339.4117), and the smallest was the BC-M strain (I = 248.2108). It showed that beta-cypermethrin reduced the growth capacity of the BC-M strain. At the same time, the growth capacity of the BC-H strain population trend index was higher than the SS strain, which indicated the enhanced potential growth capacity of the population (Table 5).

As shown in Table 6, the net reproductive rates (*R*_0_) of the SS, BC-H, and BC-M strains were 327.89, 299.76, and 322.61, the intrinsic rates (*r_m_*) of increase were 0.1966, 0.2180, and 0.2010, and the finite rates of increase (λ) were 1.2173, 1.2436, and 1.2228, respectively. There were no significant differences in *R*_0_, *r_m_*, and *λ* among the three strains. The generation duration of the SS strain (29.50 d) was significantly longer than that of the BC-M strain (26.50 d), while the BC-H strain (28.44 d) was not significantly different from either of the two (df = 2, F = 3.89, *p* = 0.082 > 0.05). Analysis of the results in terms of population growth showed that the relative fitness of the resistant strains compared to the SS strain was 0.9142 and 0.9839, respectively, which showed a reduction, but the difference is not significant.

### 4.4. Annotation Results of the SSH Library of the Beta-Cypermethrin-Resistant Strains of the Oriental Fruit Fly

#### 4.4.1. Distribution of SSH Library Data

**Figure 3:**
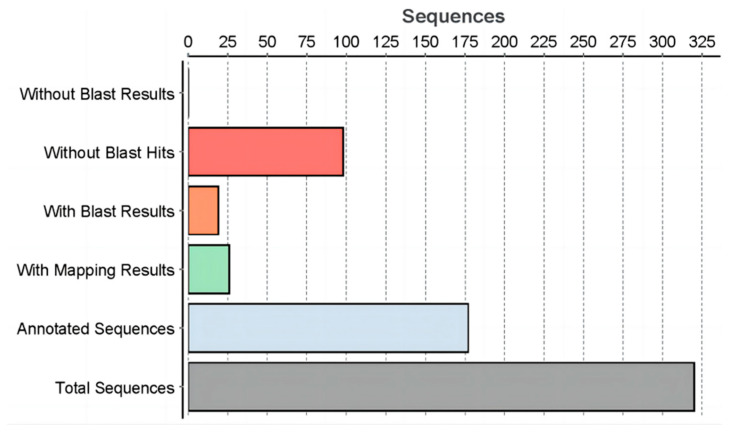
Distribution of SSH library data of the beta-cypermethrin-resistant strains of the oriental fruit fly.

The sequence fragment length analysis showed that 91.9% of the SSH library sequences from the beta-cypermethrin-resistant strains of the oriental fruit fly were within the range of 150 bp–800 bp. The alignment with Blast2go 5.2 software and the NCBI network database showed that 177 sequences from the beta-cypermethrin SSH library were annotated, while 26 sequences obtained mapping without annotation, and 19 sequences obtained blast-only results. A total of 98 sequences with no matches yielded homologous sequences and 89.1% of the sequences were matched. Homologous sequences with identity between 55% and 85% accounted for 73.4% of the library sequence (Figure 3).

#### 4.4.2. Distribution of Species in SSH Library Sequences

**Figure 4:**
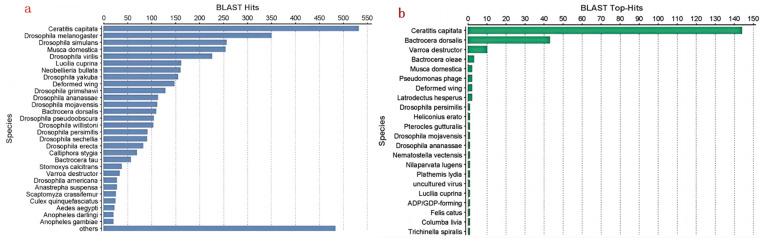
Distribution of species in SSH library sequences. (**a**) Distribution of species with homology in SSH library sequences; (**b**) distribution of species with high homology in SSH library sequences.

From the results of the homology species comparison, it can be seen that the sequence comparison results were dominated by Diptera species such as the *Ceratitis capitata*, *Drosophila*, and *B. dorsalis*. (Figure 4a). As shown in Figure 4b, the results of high-homology species of the SSH library sequences showed that the most highly homologous species were *Ceratitis capitata*, *Drosophila*, Tephritidae, and other insect species like *Cricket paralysis.*

#### 4.4.3. Proportion of SSH Libraries Involved in Biological Processes

The GO annotations of the SSH library sequences of the beta-cypermethrin-resistant strains obtained a Level 2 result distribution for biological process, molecular function, and cellular component. A total of 903 GO terms were obtained, with an average of 3.212 GO terms per sequence (Table 7).

#### 4.4.4. Sequence Annotation Plot of the SSH Library for the Resistant Beta-Cypermethrin Strains

**Figure 5:**
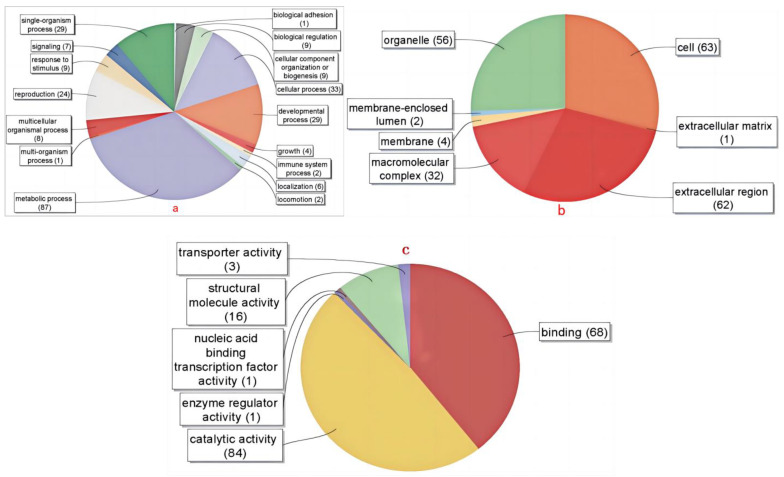
Sequence annotation plot of the SSH library for the resistant beta-cypermethrin strains. (**a**) Classification map of biological pathways (Level 2); (**b**) classification diagram of cellular components (Level 2); (**c**) classification map of molecular functions (Level 2).

From the results of the Level 2 classification of biological pathways annotated in the SSH library (Figure 5a), it can be seen that the genes in the SSH library are involved in biological processes such as the metabolic process, cellular process, developmental process, single-organism process, reproduction, and so on.

The results of the Level 2 classification of cellular components annotated by SSH library sequences (Figure 5b) showed that five major categories of genes were annotated in the SSH library sequences, namely cell (25.5%), organelle (28.2%), macromolecular complex (14.5%), membrane (1.8%) and membrane-enclosed lumen (0.9%).

The Level 2 classification of the molecular functions of the SSH library sequence annotations (Figure 5c) showed that the SSH library sequence annotations co-matched with catalytic activity (48.6%), binding activity (39.3%), structural activity (9.2%), transporter activity (1.7%), nucleic acid binding transcription factor activity (0.6%), and enzyme regulator activity (0.6%).

#### 4.4.5. Candidate Gene

By using the Blast2go 5.2 software and the NCBI web database to compare the annotations, we found that the SSH library of beta-cypermethrin-resistant strains of the oriental fruit fly annotated 51 species matches, mostly from *Ceratitis capitata*, *Drosophila*, and *B. dorsalis*. A total of 87 genes were annotated, including five beta-cytochrome P450 genes, five glutathione *S*-transferase genes, and many other genes. Resistance-related candidate genes were selected from the SSH library of the beta-cypermethrin-resistant strains of the oriental fruit fly as target genes in this study (Table 8).

### 4.5. Relative Expression of Target Genes at Different Developmental Stages of Beta-Cypermethrin-Resistant Strains of the Oriental Fruit Fly and in Different Tissues of Adults

#### 4.5.1. Quantitative Expression Results of the Target Gene across Each Insect Developmental States

**Figure 6:**
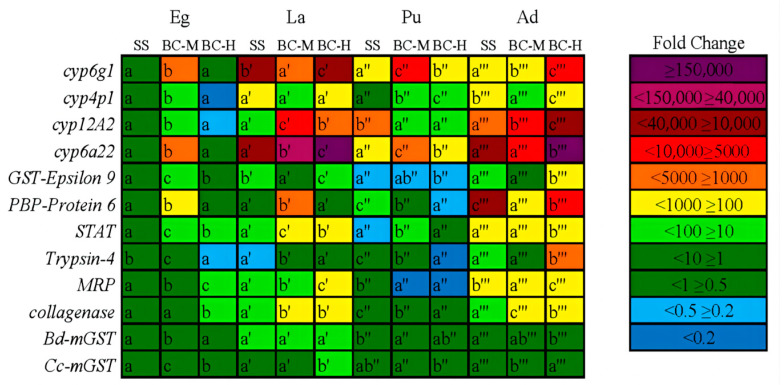
Relative expression of the target genes of the three strains of the oriental fruit fly at different developmental states. Note: Quotation marks placed to the upper right of letters indicate grouping, and different letters in the same row within a group indicate significant differences at the 0.05 level (Duncan’s method).

The expression results of the candidate genes at different developmental stages of each oriental fruit fly strains, using the eggs of SS strain as the control, are shown in Figure 4. As seen in Figure 6, the cytochrome P450 genes, *cyp6g1*, *cyp4p1*, *cyp12A2*, and *cyp6a22*, were highly expressed in larvae, pupae, and adults of all strains, with more pronounced expression especially in larvae and adults. *GST-Epsilon 9* was highly expressed in larvae and adults of all strains. All other genes except *Trypsin-4, Bd-mGST,* and *Cc-mGST* showed high expression in both adults and larvae of some strains, while *Trypsin-4* was highly expressed in adults of high resistant strains, *Bd-mGST* was upregulated in larval stages, and *Cc-mGST* was upregulated in the larval stages of high resistant strain.

Below are the candidate genes whose expression increased profoundly in upregulation with increasing pesticide resistance in high resistant strain. In the egg stage, MRP genes increased 19.78 times (df = 2, F = 115.27, *p* < 0.001); *Collagenase* genes increased 15.86 times (df = 2, F = 347.67, *p* < 0.001). In the larval stage, *cyp6a22* genes increased 31,169.77 times (df = 2, F = 2721.53, *p* < 0.001); *MRP* genes increased 467.39 times (df = 2, F = 525.30, *p* < 0.001); *Cc-mGST* genes increased 20.42 times (df = 2, F = 12.78, *p* < 0.001). In the pupal stage, *cyp4p1* gene increased 27.86 times (df = 2, F = 64.79, *p* < 0.001); In the adult stage, *cyp6g1* genes increased 5066.12 times (df = 2, F = 44,705,135.10, *p* < 0.001); *cyp12A2* genes increased 23,999.32 times (df = 2, F = 1395.79, *p* < 0.001); *cyp6a22* genes increased 1,500,935.75 times (df = 2, F = 1893.047, *p* < 0.001); *GST-Epsilon 9* genes increased 491.72 times (df = 2, F = 834.986, *p* < 0.001); *STAT* genes increased 899.74 times (df = 2, F = 1189.50, *p* < 0.001); *Trypsin-4* genes increased 1032.47 times (df = 2, F = 727.06, *p* < 0.001).

Combining the results of the comparisons, it was found that resistance genes were predominantly upregulated in adults and larvae. The genes upregulated were mainly cytochrome P450 and glutathione S-transferase genes, along with an upregulation of one resistance-related gene (*MRP*). Since both the actual field application of insecticide and the treatment in the present study for the selection of insecticide-resistant strains both occurred during the adult stage of the oriental fruit fly, the relative expression of seven genes, including *cyp6g1*, *cyp4p1*, *cyp6a22*, *cyp12A2*, *GST-Epsilon9*, *MRP,* and *Trypsin-4*, in various tissues of the adult insect will be further analyzed based on the results of the expression analysis above.

#### 4.5.2. Quantitative Expression Results of Target Genes in Different Tissues

**Figure 7:**
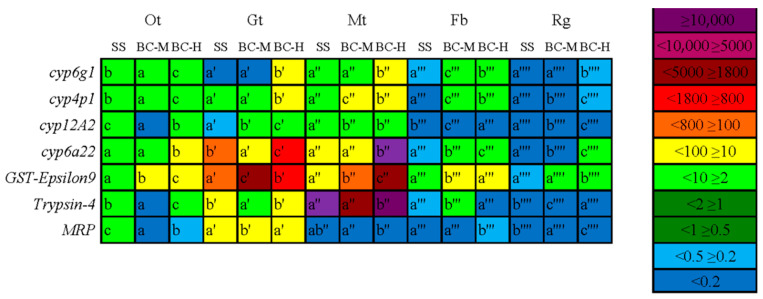
Relative expression of target genes in different tissues of three strains of adult oriental fruit fly. Note: Ot: other tissues; Gt: midgut; Mt: Malpighian tubules; Fb: fat body; Rg: reproductive gland.

The midgut and the Malpighian tubules exhibited the most significant upregulation with six genes showing upregulated expressions, while four genes were upregulated in the other tissues and the fat body. In the reproductive glands, only the *GST-Epsilon 9* of the high resistant strain was upregulated (Figure 7).

Those whose expression was significantly upregulated with increasing drug resistance are listed below.

In the midgut, the *cyp6g1* gene was upregulated 24.94 times (df = 2, F = 9805.41, *p* < 0.001); The *cyp6a22* gene was upregulated 824.23 times (df = 2, F = 4102.08, *p* < 0.001). In the Malpighian tubules, the *cyp6g1* gene was upregulated 27.45 times (df = 2, F = 739.72, *p* < 0.001); The *cyp6a22* gene was upregulated 7969 times (df = 2, F = 216.30, *p* < 0.001); The *GST-Epsilon 9* gene was upregulated 1902.25 times (df = 2, F = 6576.77, *p* < 0.001); The *Trypsin-4* gene was upregulated 38909.49 times (df = 2, F = 824.14, *p* < 0.001). In the other tissues, the *GST-Epsilon 9* gene was upregulated 51.39 times (df = 2, F = 823.49, *p* < 0.001).

## 5. Discussion

Chen’s research found no differences in the developmental periods of eggs, larvae, and pupae between the trichlorfon-resistant strain and the sensitive strain, which is consistent with the results of this experiment [36]. Similarly, in this study, we found no differences in the developmental stages of eggs, larvae, and pupae between the beta-cypermethrin medium resistance strain and the SS strain. There was also no change in the lifespan of males in the beta-cypermethrin-resistant strain compared to the SS strain. However, the results of other biological characteristics showed that the BC-M strain had a remarkably lower egg hatching rate, larval/pupal rate, and pupal instar rate, and a shorter pre-oviposition stage, while the average adult and female lifespans of the BC-H strain were substantially longer than those of the SS strain. Survival rate curves showed that adults of all strains could reach the average lifespan under the ideal laboratory environment. The daily egg production dynamics of BC-M and BC-H strains were relatively consistent and considerably lower than those of the sensitive strains, which indicated that the fecundity of the resistant strains was lower. The population trend index of beta-cypermethrin-resistant strains is lower than that the sensitive strain, while the high resistant strain has a higher index than the sensitive strain. This indicates that the effect of beta-cypermethrin on the population of orange fruit fly showed a phenomenon of low inhibition and high growth. The relative fitness of the beta-cypermethrin-resistant strain was lower than that of the sensitive strain, showing poor reproduction of the resistant populations.

Varying degrees of resistance to beta-cypermethrin have emerged in several field populations of orange fruit fly in South China [37,38]. The main mechanisms of insecticide resistance include reduced epidermal penetration, with some studies finding that epidermal competition for endogenous RNAs regulates insecticide penetration and resistance [39,40]; deciphering metabolic enhancement since metabolic genes play a major role in the entry of insecticides into the insect’s body [41,42]; reduced target gene sensitivity, through which insect nerve receptors and ion channels control insect targets [40] and target genes used in pest control [43]; chelation, in which carboxylesterase binds with high affinity to insecticides [44,45]; chemosensory proteins that can bind pesticides in large quantities and contribute to insect resistance [46]; metabolism of harmful substances by insect symbiotic bacteria that can decipher harmful chemicals and enhance immunity [47]; and insect behavioral resistance, which includes behavioral avoidance and a reduction in exposure to insecticides [48,49]. Understanding the mechanism of resistance to beta-cypermethrin in orange fruit fly will be helpful to provide a theoretical basis for the development of effective dosing strategies.

In this study, we obtained high-homology matches with 51 species and 149 genes from the SSH library, such as the cytochrome P450 gene, glutathione *S*-transferase gene, multi-resistance-associated protein gene, trypsin gene, etc. The annotated cytochrome P450 genes and glutathione *S*-transferase genes were identified as the main deciphered metabolic genes, which perfectly matched the representative resistance genes screened by Shen’s transcriptome study [50]. From the quantitative expression results, the genes that showed significant upregulation at higher multiplicity with the enhancement of insecticide resistance during the various developmental stages of the oriental fruit fly, *cyp6g1*, *cyp6a22*, and *GST-Epsilon 9,* and Trypsin-4, were upregulated in the midgut and the Malpighian tubules in response to the increase in insecticide resistance. This is consistent with the results of fluorescence quantification of 11 mitochondrial-encoded genes in Jiang’s research, which found expression in the midgut at a high level [51]. The *cyp6a22* gene also showed upregulated expression in the fat body of a high resistant strain. Hu studied seventeen GST genes in insecticide-resistant orange fruit fly and showed that six genes were overexpressed in the Malpighian tubules of the resistant strain, four were overexpressed in the fat body, and only three genes were highly expressed in the midgut, with the *GST-Epsilon 9* gene being highly expressed [52].

In this thesis, based on the resistance-related genes obtained from the SSH library, the expression of candidate genes in different tissues at different developmental stages was investigated in strains with different degrees of resistance cultivated in the laboratory over many generations. The study focused on three P450 genes, *cyp6g1*, *cyp6a22,* and *GST-Epsilon 9*, and a trypsin-like serine protease gene, *Trypsin-4,* as candidate genes for the development of resistance to beta-cypermethrin in orange fruit flies.

## 6. Conclusions

In this study, we investigated the biological dynamics of different resistances to beta-cypermethrin in the oriental fruit fly. An SSH library was constructed and annotated and analyzed for the oriental fruit fly SSH library. When the authors performed the annotation, the similarity only considered the exact matching positions and did not consider the gaps and insertions. This is because gaps and insertions affect other aspects of similarity and comparison quality such as gap coverage, gap length, identity, E-value, score, coverage, etc. Possible candidate genes in the SSH library were selected for quantitative analysis. However, the study was limited to laboratory conditions, and specific field applications should take into account the biological dynamics of the oriental fruit fly against beta-cypermethrin. In summary, the study of beta-cypermethrin in this paper contributes to a more comprehensive understanding of the effects of insecticides on individual insects and populations. Our results provide support for field use, which can be utilized for field control. In addition, our data provide new experimental data for the molecular mechanism of beta-cypermethrin resistance. Additionally, these candidate genes will serve as potential targets for further functional verification of beta-cypermethrin resistance-related genes, which will be beneficial to the resolution of the resistance mechanism.

## Figures and Tables

**Table 1:** Sequences of primers for real-time PCR.

Gene	Primer Sequence (F/R)	Product Size (bp)	Amplification Efficiency (%)	Correlation Coefficient (R^2^)
*α-tub*	5′-CGCATTCATGGTTGATAACG-3′ 5′-GGGCACCAAGTTAGTCTGGA-3′	184	103.6	0.998
*cyp6g1*	5′-ATCGCCTTTTTCGTCCCCAA-3′ 5′-GCGTTGCTTTGCTCTCCTCT-3′	127	95.8	0.993
*cyp4p1*	5′-CCTTTTTACCACATAATCACCGCT-3′ 5′-AGGCAGGAGTCAGCATTTTTC-3′	172	89.3	0.997
*cyp12A2*	5′-GATTGGGCGAAAATACGGAC-3′ 5′-AGTCATCGGGCATCTCGTTT-3′	154	89.5	0.998
*cyp6a22*	5′-CCGCTCACTGGCAATCTCTT-3′ 5′-TGTCGGGTGCGTTTTTAGTTT-3′	172	91.6	0.999
*GST-Epsilon 9*	5′-AGCGAACAGCCACAAG-3′ 5′-ACGAGTGCTTCCAAACC-3′	270	120.6	0.971
*Bd-mGST*	5′-CGCCACCATTAGCAACATTT-3′ 5′-GCCCAGAAAAGAAAGCAACAGA-3′	115	103.8	0.999
*Cc-mGST*	5′-CCCGAGGATTTGATGGACAA-3′ 5′-CGTAGAGGAAGCCAATGATGA-3′	127	99.7	1.000
*MRP*	5′-CGGTATGGTGTCGTCGGTTAT-3′ 5′-AGGCGTTCGGTCAATCTCTC-3′	225	85.1	0.975
*PBP-Protein 6*	5′-TTTGACCGCTTGGATTGCCT-3′ 5′-CACTTGGGTTCGCCACCTTT-3′	125	110.1	0.998
*STAT*	5′-ATCCACGACAACAAACGCTC-3′ 5′-GCACCACAACAACTATGGGT-3′	145	100.3	0.987
*Trypsin-4*	5′-CTACAACCCACTCACCTCCG-3′ 5′-GGCACACCAGCACATAGCA-3′	276	91.5	1.000
*collagenase*	5′-GTCAGTGCCATTTTCGCCTT-3′ 5′-ATTGCGGGTTTCGGCTTCAC-3′	162	105.3	0.985

**Table 2:** Life table attributes of oriental fruit fly.

Strains	Eggs			Larvae			Pupae		
	Developmental Duration (h)	Developmental Rate	Hatching Rate (%)	Developmental Duration (h)	Developmental Rate	Pupal Rate (%)	Developmental Duration (h)	Developmental Rate	Emergence Rate (%)
SS	45.83 ± 3.60 a	0.0225 ± 0.0018 a	88.33 ± 2.99 ab	123.17 ± 1.01 a	0.0081 ± 0.0001 a	95.18 ± 1.47 b	231.78 ± 3.99 a	0.0043 ± 0.0001 a	97.93 ± 1.01 b
BC-H	53.17 ± 1.78 a	0.0189 ± 0.0006 a	93.67 ± 2.03 b	124.50 ± 4.14 a	0.0081 ± 0.0002 a	96.66 ± 1.63 b	241.67 ± 5.03 a	0.0042 ± 0.0001 a	95.76 ± 1.15 ab
BC-M	45.00 ± 5.57 a	0.0230 ± 0.0032 a	82.67 ± 2.91 a	122.67 ± 2.03 a	0.0082 ± 0.0001 a	84.79 ± 4.77 a	234.50 ± 1.34 a	0.0043 ± 0.0000 a	89.27 ± 4.92 a

Notes: SS: sensitive strain, BC-H: high resistant strain to beta-cypermethrin, BC-M: medium resistant strain to beta-cypermethrin. The notation applies to all the tables and figures below. The same below. Data are presented as mean ± SE, and data in the same column followed by different letters indicate significant differences at the 0.05 level (Duncan’s method).

**Table 3:** Main biological parameters of beta-cypermethrin-resistant and -sensitive strains of the adult oriental fruit fly.

Strains	Pre-Oviposition Period (d)	Adult Longevity (d)		Fecundity (eggs/female^−1^)	Average Survival Time (d)
		Female	Male		
SS	7.33 ± 0.33 b	119.00 ± 2.00 a	124.67 ± 6.12 a	864.61 ± 21.86 a	127.33 ± 4.33 a
BC-H	7.00 ± 0.00 b	150.33 ± 9.02 b	144.00 ± 6.66 a	848.11 ± 41.78 a	150.33 ± 9.02 b
BC-M	6.00 ± 0.00 a	136.33 ± 1.67 ab	123.67 ± 7.22 a	791.14 ± 39.72 a	136.33 ± 1.67 ab

Notes: Data are presented as mean ± SE, and data in the same column followed by different letters indicate significant differences at the 0.05 level (Duncan’s method).

**Table 4:** Equations of the survival curves of the adult oriental fruit flies of three strains.

Strains	Equation	Correlation Coefficient (r)
SS	$S_{p}(t)=e^{{-(\frac{t}{67.484})}^{2.600}}$	0.9985
BC-H	$S_{p}(t)=e^{{-(\frac{t}{74.874})}^{1.734}}$	0.9930
BC-M	$S_{p}(t)=e^{{-(\frac{t}{70.382})}^{2.119}}$	0.9990

**Table 5:** Life table of experimental populations of beta-cypermethrin-resistant and -sensitive strains of the oriental fruit fly.

Developmental Stage	Impact Factor	Survival Rate		
		SS	BC-H	BC-M
Eg	Non-hatching	0.8833 ± 0.0299 ab	0.9367 ± 0.0203 b	0.8267 ± 0.0291 a
La	Recessive death	0.9518 ± 0.0147 b	0.9666 ± 0.0163 b	0.8479 ± 0.0477 a
Pu	Non-emergence	0.9793 ± 0.0101 b	0.9576 ± 0.0115 ab	0.8927 ± 0.0492 a
Ad	Proportion of females	0.4768 ± 0.0245 a	0.4878 ± 0.0339 a	0.5014 ± 0.0374 a
	Probability of standard fecundity	0.4323 ± 0.0109 a	0.4241 ± 0.0209 a	0.3956 ± 0.0199 a
	Standard fecundity	2000	2000	2000
Population trend index (*I*)		339.4117	358.7071	248.2108

Notes: Data are presented as mean ± SE, and data in the same line followed by different letters indicate significant differences at the 0.05 level (Duncan’s method). Eg: egg; La: third instar larva; Pu: pupae; Ad: adult. The same below.

**Table 6:** Population parameters and relative fitness of the susceptible and beta-cypermethrin-resistant strains of the oriental fruit fly.

Strains	Net Reproductive Rate (*R*_0_)	Intrinsic Rate of Increase (*r_m_*)	Finite Rate of Increase (*λ*)	Average Generation Duration (*T*)	Relative Fitness (*R_f_*)
SS	327.89 ± 3.07 a	0.1966 ± 0.0051 a	1.2173 ± 0.0062 a	29.50 ± 0.75 b	1.0000
BC-H	299.76 ± 13.50 a	0.2010 ± 0.0082 a	1.2228 ± 0.0101 a	28.44 ± 1.00 ab	0.9142
BC-M	322.61 ± 16.35 a	0.2180 ± 0.0036 a	1.2436 ± 0.0044 a	26.50 ± 0.47 a	0.9839

Notes: Data are presented as mean ± SE, and data in the same column followed by different letters indicate significant differences at the 0.05 level (Duncan’s method).

**Table 7:** Proportion of annotated sequences contributing to bioprocesses in the SSH library of beta-cypermethrin-resistant strains of the oriental fruit fly.

Biological Process	Proportion of Annotated Sequences of Beta-Cypermethrin-Resistant Strains
Metabolic process	33.5%
Cellular process	12.7%
Single-organism process	11.2%
Developmental process	11.2%
Response to stimulus	3.5%
Reproduction	9.2%
Locomotion	0.8%
Localization	2.3%
Cellular component organization or biogenesis	3.5%
Growth	1.5%
Immune system process	0.8%
Signaling	2.7%
Multicellular organismal process	3.1%
Biological regulation	3.5%
Biological adhesion	0.4%
Multi-organism process	0.4%

**Table 8:** Candidate genes for resistance to beta-cypermethrin in the oriental fruit fly.

Protein Family	Genetics	Conservative Area	Matched Species
Cytochrome P450	*cytochrome P450 6g1*	p450/cpyx	*Ceratitis capitata*
	*cytochrome P450 6a22*	p450/cpyx	*Ceratitis capitata*
	*cytochrome P450 4p1*	p450/cpyx	*Ceratitis capitata*
	*cytochrome P450 12A2*	p450/cpyx	*Ceratitis capitata*
Glutathione S-transferase	*epsilon class 9*	C-terminal, alpha helical domain of class delta and epsilon glutathione S-transferases	*Bactrocera drosalis*
	*microsomal glutathione S-transferase*	MAPEG (membrane-associated proteins in eicosanoid and glutathione metabolism) family	*Ceratitis capitata*
	*microsomal glutathione S-transferase*		*Bactrocera drosalis*
Pheromone-binding protein related protein	*protein 6*	PBP/GOBP family, insect pheromone/odorant binding protein domains	*Ceratitis capitata*
Trypsin-like serine protease	*trypsin-4*	Trypsin-like serine protease	*Ceratitis capitata*
	*collagenase*	Trypsin-like serine protease	*Ceratitis capitata*
Multi-insecticide resistance protein	*ATP-binding cassette domain*	ATP-binding cassette domain	*Ceratitis capitata*
Signal transducer and transcription activator	*signal transducer and transcription activator*	STAT protein, DNA-binding domain/all-alpha domain	*Ceratitis capitata*

## Data Availability

The data presented in this study are available on request from the corresponding author.
